# Unlocking the power of AI models: exploring protein folding prediction through comparative analysis

**DOI:** 10.1515/jib-2023-0041

**Published:** 2024-05-27

**Authors:** Paloma Tejera-Nevado, Emilio Serrano, Ana González-Herrero, Rodrigo Bermejo, Alejandro Rodríguez-González

**Affiliations:** ETS Ingenieros Informáticos, 16771 Universidad Politécnica de Madrid, Madrid, Spain; Centro de Tecnología Biomédica, 16771 Universidad Politécnica de Madrid, Pozuelo de Alarcón, Madrid, Spain; 54446 Margarita Salas Center for Biological Research (CIB-CSIC), Spanish National Research Council, Madrid, Spain

**Keywords:** protein structure prediction, protein folding estimation, protein conformation analysis, artificial intelligence models, deep learning models, root-mean-square deviation

## Abstract

Protein structure determination has made progress with the aid of deep learning models, enabling the prediction of protein folding from protein sequences. However, obtaining accurate predictions becomes essential in certain cases where the protein structure remains undescribed. This is particularly challenging when dealing with rare, diverse structures and complex sample preparation. Different metrics assess prediction reliability and offer insights into result strength, providing a comprehensive understanding of protein structure by combining different models. In a previous study, two proteins named ARM58 and ARM56 were investigated. These proteins contain four domains of unknown function and are present in *Leishmania* spp. ARM refers to an antimony resistance marker. The study’s main objective is to assess the accuracy of the model’s predictions, thereby providing insights into the complexities and supporting metrics underlying these findings. The analysis also extends to the comparison of predictions obtained from other species and organisms. Notably, one of these proteins shares an ortholog with *Trypanosoma cruzi* and *Trypanosoma brucei*, leading further significance to our analysis. This attempt underscored the importance of evaluating the diverse outputs from deep learning models, facilitating comparisons across different organisms and proteins. This becomes particularly pertinent in cases where no previous structural information is available.

## 1 Introduction

The transformation of a polypeptide chain into its biologically active 3D structure, known as protein folding, holds immense importance in diverse applications like drug design, understanding protein–protein interactions, and gaining insights into the molecular mechanism of diseases.

Traditional methods for protein structure determination, like X-ray, NMR (Nuclear Magnetic Resonance), and EM (Electron Microscopy), can be both expensive and time-consuming due to their resource and expertise demands [1, 2]. Protein folding is a complex process, presenting challenges such as numerous potential conformations, the crowded cellular environment, and navigating a complex energy landscape to attain the final structure. However, the arrival of deep learning approaches for protein folding prediction has transformed the field [3], allowing *in silico* predictions that can be experimentally validated, introducing a novel and promising approach to traditional biochemistry.

The main objective of the Critical Assessment of Protein Structure Prediction (CASP) experiments is to evaluate the current state of the art in protein structure prediction, monitor the progress made so far, and identify areas where future efforts can be most productively focused. Bi-annually, the CASP meeting reveals that deep learning methods like AlphaFold, Rosetta, RoseTTAFold and trRosetta prove to be more effective than traditional approaches that explicitly model the folding process [4, 5].

AlphaFold2, a novel computational approach, has demonstrated the ability to predict protein structures with exceptional accuracy close to experimental results in most cases. Submitted as AlphaFold2 to the CASP14 competition, it employs a distinct model from the previous AlphaFold system used in CASP13 [6]. Moreover, a multimer version of AlphaFold has been released, enabling scientists to forecast protein complexes and identify protein–protein interactions. Utilizing AlphaFold-Multimer results in enhanced accuracy when predicting multimeric interfaces compared to the input-adapted single-chain AlphaFold, while preserving a high level of accuracy within individual chains [7].

To fully harness these methods, researchers need access to robust computing resources, prompting the development of alternative platforms. One such platform is ColabFold, which combines the swift homology search of MMseqs2 with AlphaFold2, enabling accelerated prediction of protein structures and complexes [8]. Additionally, the Galaxy server has incorporated AlphaFold 2.0 for bioinformatics analysis since early 2022. Collaborating to accelerate scientific research, DeepMind and EMBL’s European Bioinformatics Institute (EMBL-EBI) created AlphaFold DB, providing open access to over 200 million protein structure predictions [6, 9]. Another community project, CAMEO (Continuous Automated Model EvaluatiOn), was developed by the Computational Structural Biology Group at the SIB Swiss Institute of Bioinformatics and the Biozentrum of the University of Basel. CAMEO continuously assesses the accuracy of protein prediction servers based on known experimental structures from the Protein Data Bank (PDB). Users can submit multiple models for a target protein, and CAMEO evaluates up to 5 models, including continual assessment of Robetta [10], a protein prediction service.

Recently, ESMFold has been introduced as a rapid and precise sequence-to-structure predictor, achieved by training transformer protein language models. This breakthrough enabled the creation of the ESM Metagenomics Atlas, containing a vast repository of over 600 million metagenomics proteins, making it a valuable resource for protein structure prediction [11].

Deep learning models achieve impressive accuracy in diverse tasks, but their predictions may vary [12–15]. To ensure reliability, careful evaluation and interpretation are essential. This involves selecting suitable, understanding the model’s assumptions and limitations, and considering their impact on predictions.

## 2 Metrics and scores

Understanding the outputs of deep learning models can be challenging. Common metrics help evaluate models, and the choice depends on the application. Interpreting machine learning predictions is an iterative process, involving ongoing analysis, identifying weakness, and refining the model’s parameters to build mode accurate and reliable models for diverse applications.

The GDT (Global Distance Test) metric measures similarity between protein structures with the same amino acid sequence but different tertiary structures. It is used to compare predictions with experimentally determined structures, and the GDT_TS score is more accurate than the root-mean-square deviation (RMSD) metric. GDT evaluates alpha carbon atoms of amino acid residues within a specific distance cutoff from the experimental structure. This metric is essential in the CASP experiment, which assesses modelling techniques and identifies deficiencies [16].

The LDDT (Local Distance Difference Test) score evaluates stereochemistry plausibility by measuring local differences between all atoms in a model, disregarding their superposition [17]. AlphaFold2 provides the predicted quality of a protein using a per-residue pLDDT score, enabling assessment of intra-domain confidence [6]. The per-residue pLDDT score indicates the predicted quality of a protein, ranging from 0 to 100, with higher scores indicating higher quality predictions. Residues with pLDDT scores above 90 are considered reliable, 70–90 scores are less reliable, and below 50 scores indicate low quality. AlphaFold2 calculates pLDDT by comparing predicted distances to a reference set of experimentally determined protein structures at the residue level, reflecting the similarity between predicted and reference structures.

The Predicted Aligned Error (PAE) metric is used by the AlphaFold system to assess the quality of predicted protein structures. PAE measures the average distance between the predicted and true residue positions after aligning both structures using a variant of the Kabsch algorithm. PAE is particularly useful for assessing confidence between domains or chains, with scores below a certain threshold identifying challenging or deviating regions in the protein’s predicted structure. Additionally, the template modelling score (TM-score) is developed for automated evaluation of protein structure template quality [18]. The predicted TM-score (pTM-score) considers the probability of each residue being resolved, weighting their contribution accordingly.

Various methods are routinely employed to compare computational models with experimental data in modelling assessments. Among these, RMSD stands out as the most frequently utilized quantitative measure for assessing the similarity between two superimposed sets of atomic coordinates, typically presented in angstroms. Global RMSD, calculated based on positional distance, is commonly employed to evaluate overall protein structure similarity [19].

In this study, we investigated two proteins with four domains of unknown function (DUF1935). A domain of unknown function (DUF) refers to a protein domain whose specific function has not been characterized. These groups of families are brought together in the Pfam database, labelled with the prefix DUF followed by a number. Pfam serves as a large collection of protein types and families, shown as alignments of multiple sequences and as models using profile hidden Markov [20]. Efforts within structural genomics initiatives have been made to decipher the function of DUFs by determining their three-dimensional structures. ARM58 has been identified in *Leishmania* species and serves as an antimony resistance marker [21, 22]. ARM56 has orthologs in *Trypanosoma* spp. and shows differences in antimony tolerance between the promastigotes and amastigotes forms in *Leishmania* [22, 23]. The orthologue of ARM56 in *Trypanosoma cruzi* and *Trypanosoma brucei* are also included in the analysis. A reference strain for each species was selected according to the information in the TriTrypDB [24]. An initial attempt was made to predict the protein structures for the sequences of ARM58 and ARM56 in *Leishmania infantum*. A comparison was carried out among the outputs and metrics using AlphaFold2, ColabFold, tr-Rosetta, tr-RosettaX-Single, RoseTTAFold, and ESMFold. Subsequently, a comparative analysis was conducted using different outputs generated by AlphaFold2 for the protein sequences in *Leishmania donovani* and *Leishmania major*. The study concluded with a comparable contrast using the protein sequences of the ortholog of ARM56 in *T. cruzi* and *T. brucei*. The study aimed to assess whether comparing different models and sequences could yield additional insights from the protein structure, striving to optimize various methods and identify the most effective approach in utilizing deep learning techniques for protein structure prediction. These techniques can serve as both routine and complementary tools, offering guidance and accelerating research.

## 3 Materials and methods

**Figure 1:**
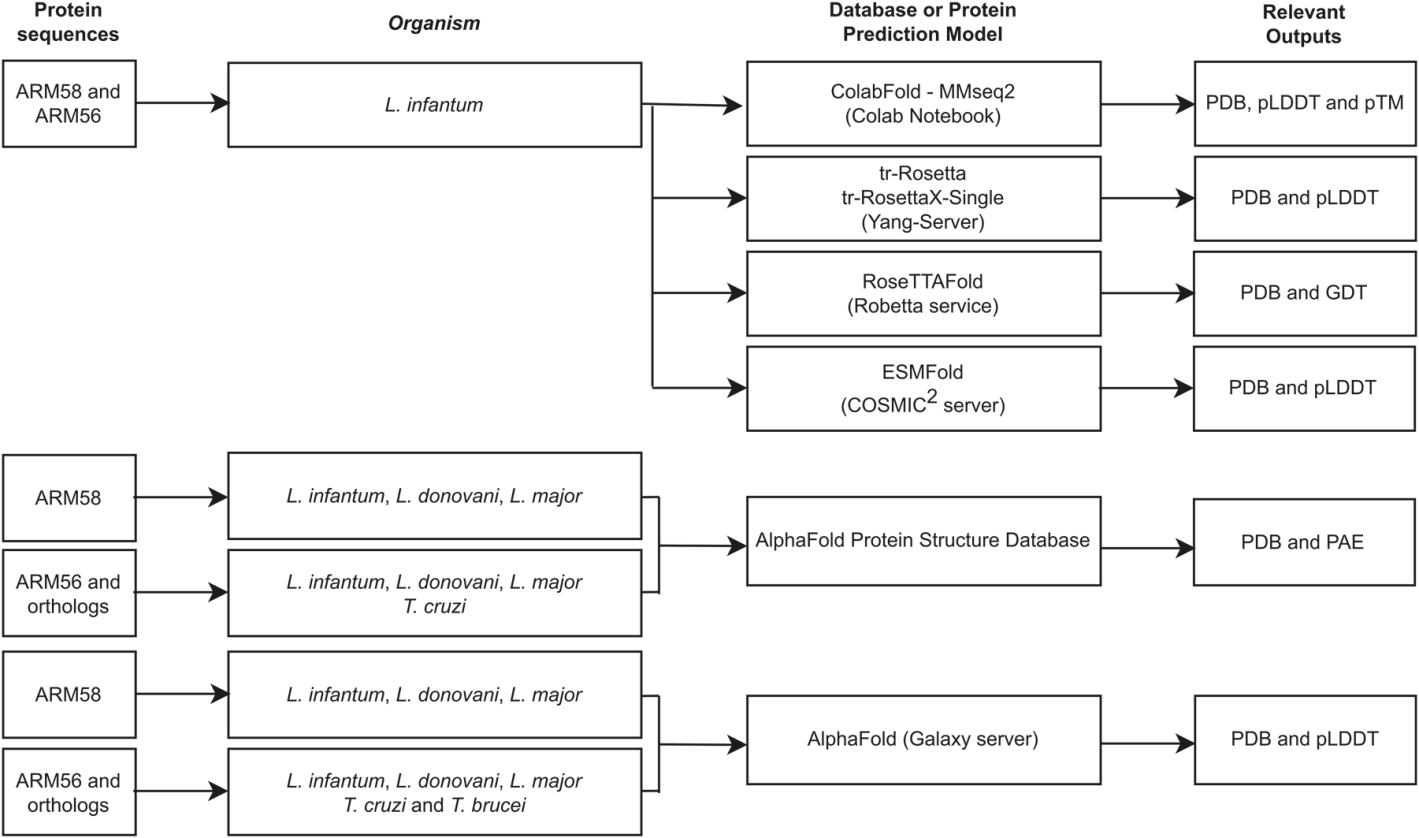
Diagram illustrating the summary of the workflow performed in the study. It encompasses the searched sequences for ARM58 and ARM56, along with the orthologs. Subsequently, the protein sequences or names were utilized to download or predict the protein structures using various available models. The relevant outputs were then considered for protein comparison. Finally, the protein sequences underwent relaxation, as described in the material and methods section.

ARM58 and ARM56 are encoded by the genes LINF_340007100 and LINF_340007000 in *L. infantum*, respectively. The protein sequences for ARM58 in *L. donovani* and *L. major* correspond to the protein products from the genes encoding for LdBPK_340220.1 and LmjF.34.0200, respectively. The protein sequences for ARM56 in these species correspond to the protein products from the genes encoding for LdBPK_340210.1 and LmjF.34.0190. The ortholog gene for ARM56 in *T. cruzi* is TcCLB.506407.50 (CL Brener Esmeraldo-like) and Tb927.10.2610 in *T. brucei brucei* (TREU927). The protein sequences contain four domains of unknown function (DUF1935), which were downloaded in FASTA format from TriTrypDB [24] and UniProt [25]. Predicted AlphaFold protein structures for ARM58 and ARM56 were also downloaded from the Protein Structure Database [6, 9], including the PDB file and the predicted aligned error. The Galaxy server was used to generate AlphaFold 2.0. predictions for both proteins. It was also used ColabFold v1.5.1 [8] which employs MMseqs2 and HHsearch for sequence alignments and templates. The protein structure prediction service Robetta [10] was used to obtain the inference using RoseTTAFold [4]. Additionally, the trRosetta server [26, 27] was used for protein structure prediction by transform-restrained Rosetta. Also, in the analysis, the trRosettaX-Single does not utilize homologous sequences and templates [28]. The COSMIC^2^ science gateway [29] was employed to use ESMFold due to its availability v1.0.3 [11]. Finally, the colab notebook relax_amber [30] was used to relax the structures using amber, with the outputs labelled as unrelaxed. Protein predictions were visualized using UCSF ChimeraX v1.6.1 [31]. The experimental workflow employed in this study is summarized, providing an overview of the key steps and methodologies utilized (Figure 1).

To quantify the similarity between protein structures, the RMSD was calculated using ChimeraX. In the alignment process, the secondary structure weight was set to 0.3 to facilitate RMSD calculation between all pairs within the two sequences. RMSD serves as a measure of the average distance between corresponding atoms, typically the backbone atoms, of aligned or superimposed molecules.

## 4 Results

**Figure 2:**
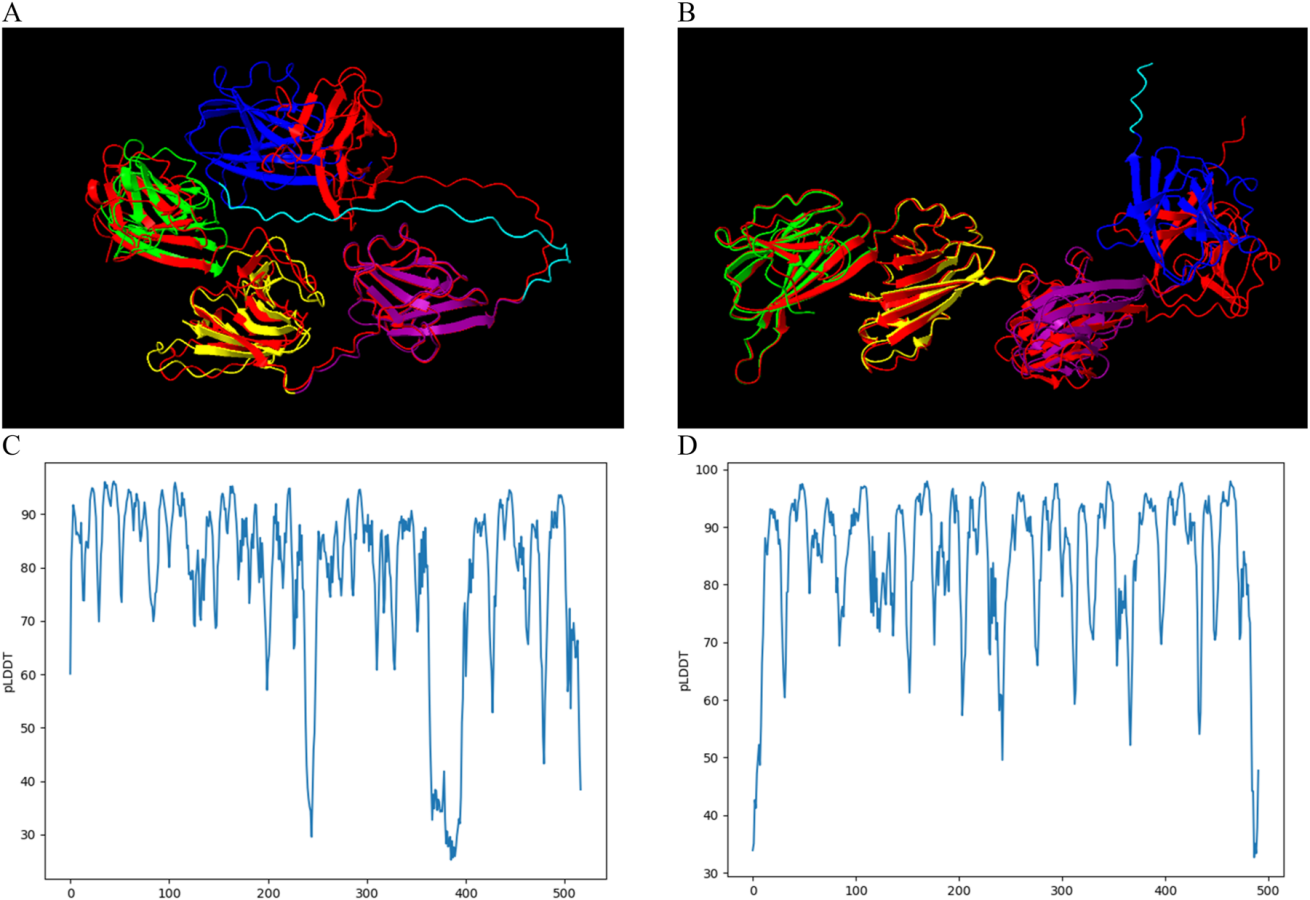
Protein structure prediction for ARM58 and ARM56 in *Leishmania infantum*. (A) The model for ARM58 from AlphaFold DB is coloured by domains (first: green, second: yellow, third: purple, and fourth: blue), while the AlphaFold prediction’s best model (model confidence score: 78.59), obtained in Galaxy, is shown in red. (B) The model for ARM56 from AlphaFold DB is coloured by domains (first: green, second: yellow, third: purple, and fourth: blue), while the AlphaFold prediction’s best model (model confidence score: 84.19), obtained in Galaxy, is shown in red. The regions containing amino acids with uncertain positions are labelled in light blue. Protein prediction structures were visualized in ChimeraX. Predicted LDDT represented by pLDDT per residue obtained by the model from Galaxy in ARM58 (C) and ARM56 (D).

Predictions of the ARM58 and ARM56 proteins utilizing AlphaFold2 were acquired from the Protein Structure DB [6, 9]. These two proteins each consist of four domains of unknown function (DUF1935), and their sequences contain 517 and 491 amino acids, respectively. Additionally, AlphaFold2 was applied via the Galaxy server. The resulting predictions were then contrasted using ChimeraX by aligning their structures. The predicted aligned error output was used to label the domains for both proteins (Figure 2A and B). The predicted LDDT plot allows for the determination of the confidence levels of the residues (Figure 2C and D).

When comparing the predicted protein structures between two outputs, even if the model is the same and the information about the confidence of the residues is added, it can be observed that in the case of ARM58, there are two regions with low pLDDT scores (around 30). The first region corresponds to the area between the second and the third domain and the amino acid positions Pro241 and Pro249. The second region corresponds to the lining area between the third and fourth domains and encompasses the region between the amino acid positions Pro365 and Pro393 (Figure 2A, the regions containing those amino acids positions have been highlighted in light blue).

Following that, ColabFold utilizes MMseqs2 for its analysis, aiming to achieve faster protein predictions. Subsequently, an analysis of the metrics and results was conducted. The tool underwent three iterations using default parameters, leading to slight fluctuations in pLDDT and pTM scores (Table 1).

**Table 1:** Average and standard deviation of the metrics pLDDT and pTM by performing three protein structure prediction runs in ColabFold using MMseqs2 for both ARM58 and ARM56, in *Leishmania infantum*.

		ARM58	ARM56
pLDDT	Average	80.433	86.733
	Standard deviation	0.577	4.619
pTM	Average	0.410	0.486
	Standard deviation	0.003	0.004

**Figure 3:**
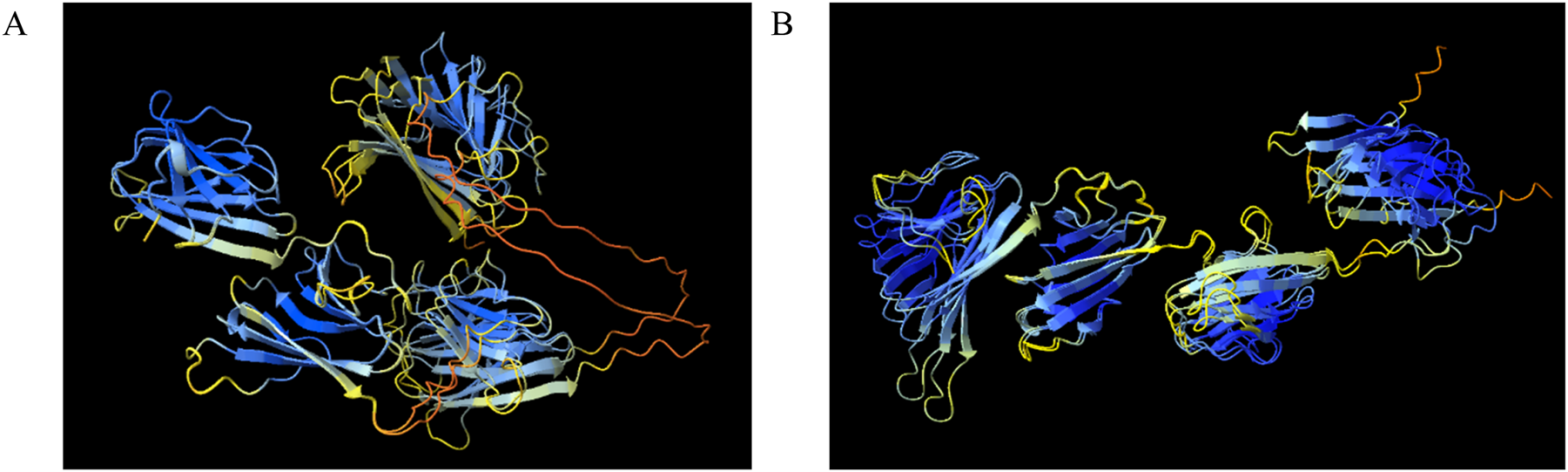
Protein structure predictions for ARM58 (A) and ARM56 (B) in *Leishmania infantum* using ColabFold with MMseqs2. Two resulting structures were coloured based on model confidence, where the dark blue represents pLDDT > 90, light blue for 90 > pLDDT > 70, yellow for 70 > pLDDT > 50 and orange for pLDDT < 50. Protein prediction structures were visualized for overlap using matchmaker in ChimeraX.

Among the three distinct executions, two protein prediction structures were chosen for further analysis, principally because of their different pLDDT and pTM scores. These selected outputs were then visualized using ChimeraX software and color-coded according to their respective pLDDT values (Figure 3).

**Figure 4:**
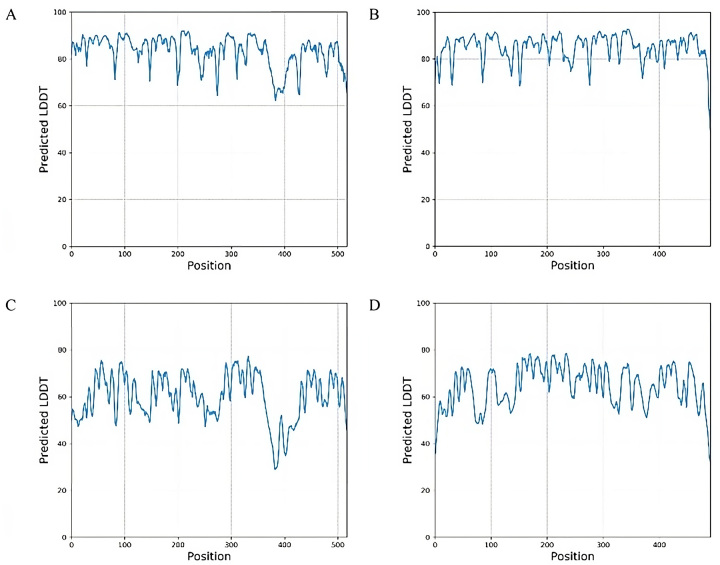
Predicted per-residue LDDT values for ARM58 and ARM56 in *Leishmania infantum*. For ARM58, predicted LDDT per amino acid position from tr-Rosetta (A) and trRosettaX-Single (C). Similarly, for ARM56, predictions from tr-Rosetta (B) and tr-RosettaX-Single (D).

Subsequently, both the trRosetta server and trRosettaX-Single were employed to predict the protein structures of ARM58 and ARM56 in *L. infantum*. The models produced by trRosetta demonstrate significant confidence, with estimated TM-scores of 0.608 for ARM58 and 0.650 for ARM56. Contrastingly, the models generated by trRosettaX-Single show reduced confidence, with TM-scores estimated at 0.275 for ARM56 and 0.266 for ARM58. The disparity in confidence is also evident in the anticipated per-residue LDDT values for the better model (Figure 4).

**Figure 5:**
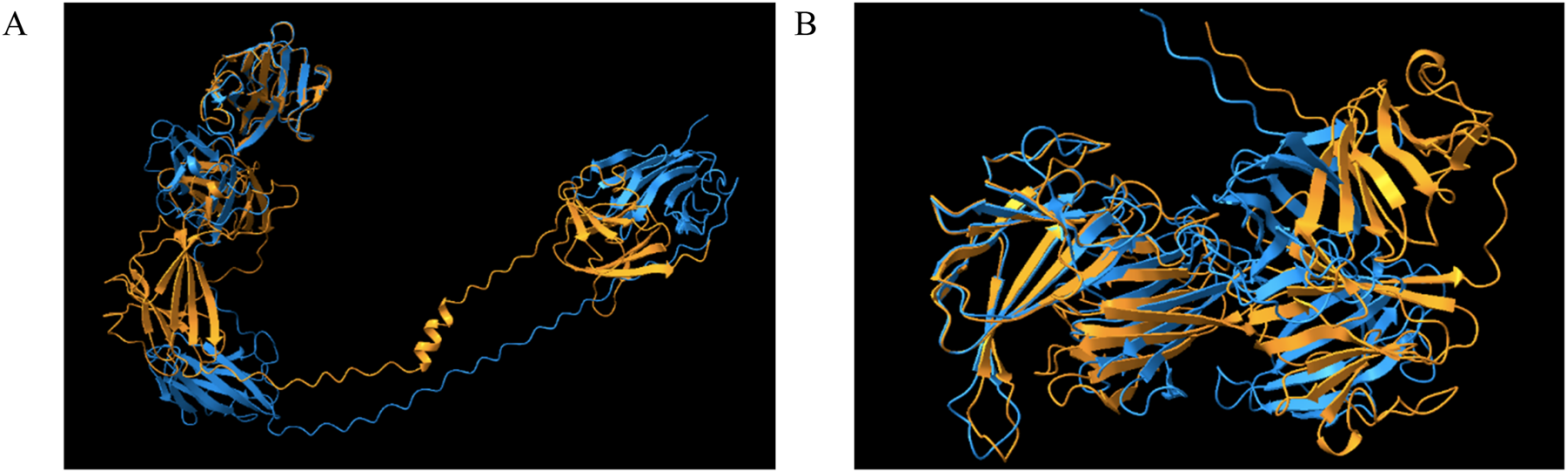
Predicted per-residue for the top models in ARM58 (A) and ARM56 (B) in *Leishmania infantum* using tr-Rosetta (in blue) and RoseTTAFold (in orange). Protein prediction structures were visualized for overlap using matchmaker in ChimeraX.

Following that, the modelling technique RoseTTAFold through the Robetta service [10] was utilized to predict the structures of ARM56 and ARM58. The predicted Global Distance Test (GDT) confidence scores for these models were 0.79 for ARM56 and 0.76 for ARM58. Notably, the tr-Rosetta and RoseTTAFold prediction models displayed slight disparities from the previously stated descriptions. To conclude, a comparison was drawn between the tr-Rosetta model and the most analogous one among the five models forecasted using RoseTTAFold (Figure 5).

**Figure 6:**
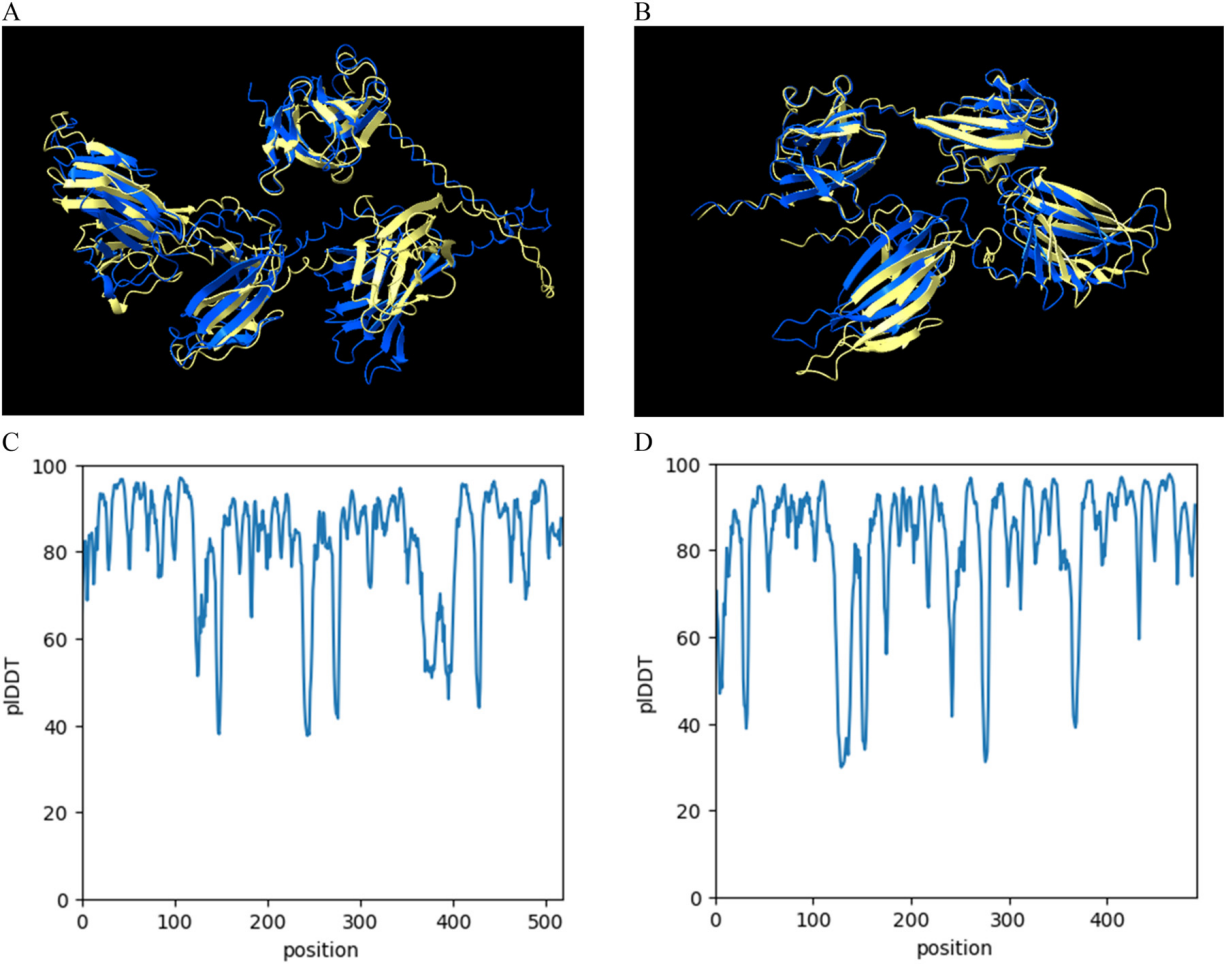
Protein structure prediction for ARM58 and ARM56 in *Leishmania infantum* using ESMFold. Model outputs for ARM58 (A) and ARM56 (B) were obtained with four recycles (yellow) and eight recycles (blue), and visualized in ChimeraX. Predicted per-residue LDDT values obtained with eight recycles for ARM58 (C) and ARM56 (D).

Next, a comparison was performed with other model known for its faster results, and the resulting variations in the outputs introduces uncertainty in the confidence of segments between the domains. To assess the impact, ESMFold was incorporated into the analysis for comparative purposes. In this context, both ARM58 and ARM56 (Figure 6) in *L. infantum* were considered. Each prediction was executed using the default number of recycles, set at 4, and the process was also replicated with an increased value of 8. The increase in the number of recycles can contribute to the generation of models with enhanced confidence.

There are not significant variations in the structural distribution when using either 4 or 8 recycles. However, the spatial distribution of the domains differs from the previously described models presented earlier. Since the structure of these proteins remains unknown through experimental analysis, and considering the accuracy of some models in comparison to previously described ones, all the models could yield different outputs due to variations, as is common with classical methods. To investigate the distinct spatial distribution resulting from the uncertain positions of these regions, sequences from two *Leishmania* spp were obtained and analysed.

**Figure 7:**
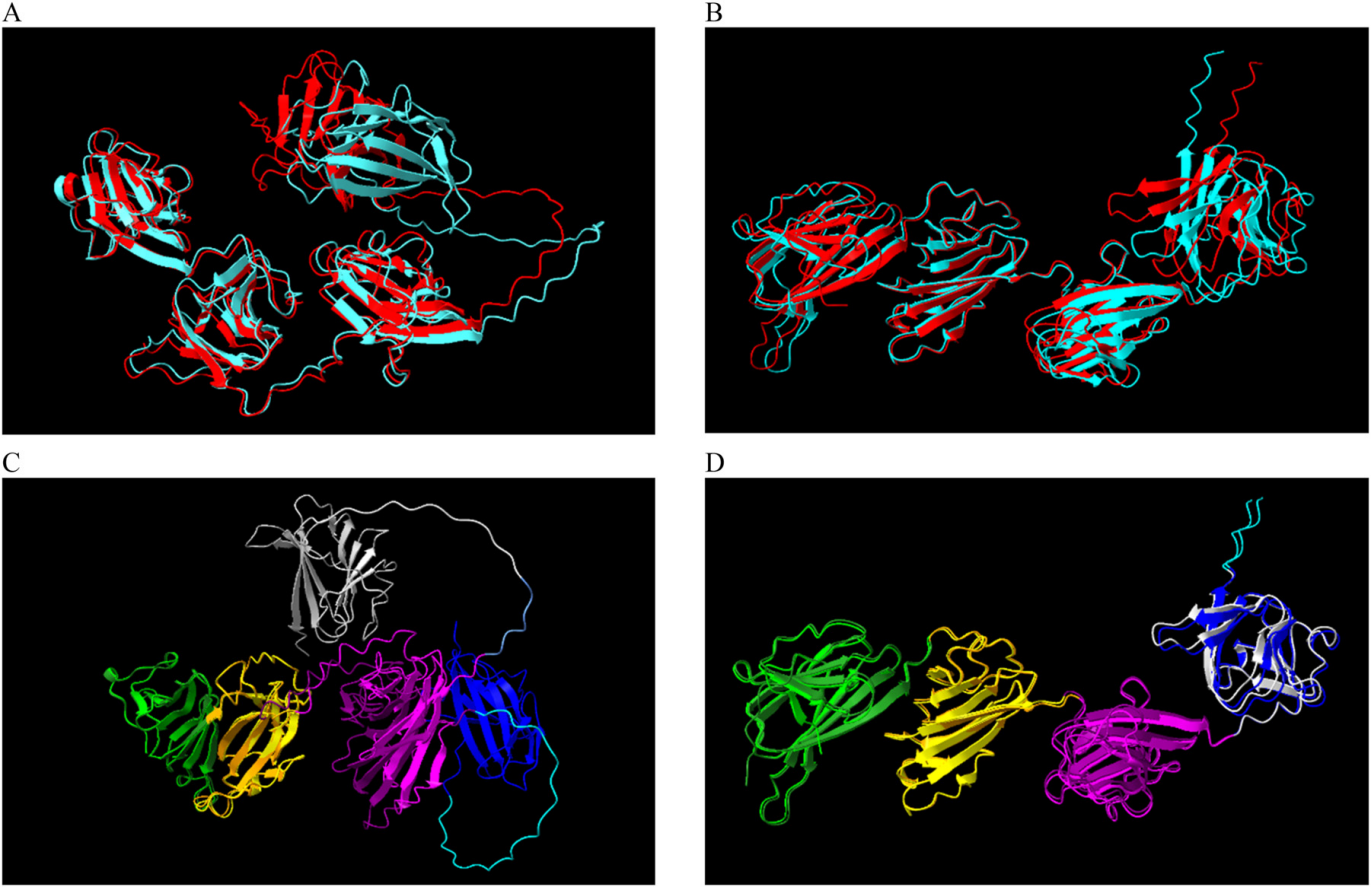
Protein structure prediction comparison for ARM58 and ARM56 in *L. donovani* and *L. major*. The models are the best predictions from AlphaFold executed in the Galaxy server and taken from the AlphaFold DB. The outputs are visualized with ChimeraX, analysing the structures with the tool matchmaker. (A) The predicted structure for ARM58 from AlphaFold in Galaxy is coloured in red (model confidence score: 78.82) and cyan (model confidence score: 79.18) in the protein sequence from *L. donovani* and *L. major*, respectively. (B) The predicted structure for ARM56 from AlphaFold in Galaxy is coloured in red (model confidence score: 86.67) and cyan (model confidence score: 84.84) in the protein sequence from *L. donovani* and *L. major*, respectively. (C) The model for ARM58 from AlphaFold DB is coloured by domains for the protein sequence in *L. donovani* (first: lime, second: yellow, third: purple, forth: blue) and in *L. major* (first: green, second: orange, third: magenta, fourth: light grey). (D) The model for ARM56 from AlphaFold DB is coloured by domains for the protein sequence in *L. donovani* (first: lime, second: yellow, third: purple, forth: blue) and in *L. major* (first: green, second: orange, third: magenta, fourth: light grey).

Next, the predicted protein structures for ARM58 and ARM56 from AlphaFold2 were evaluated using *L. donovani* BPK282A1 (518 and 491 amino acids), and *L. major* strain Friendlin (517 and 491 amino acids). This structural analysis involved comparing the outputs obtained from the execution of AlphaFold2 in Galaxy with the predicted structures from AlphaFoldDB. The goal of this examination was to determine whether comparing different species with slight variations in the sequences could provide additional insights into the spatial distribution of the proteins. Therefore, the inspection was performed with the same proteins in different species for each model. While ARM58 exhibits differences between them, ARM56 demonstrates an almost identical spatial distribution (Figure 7).

**Figure 8:**
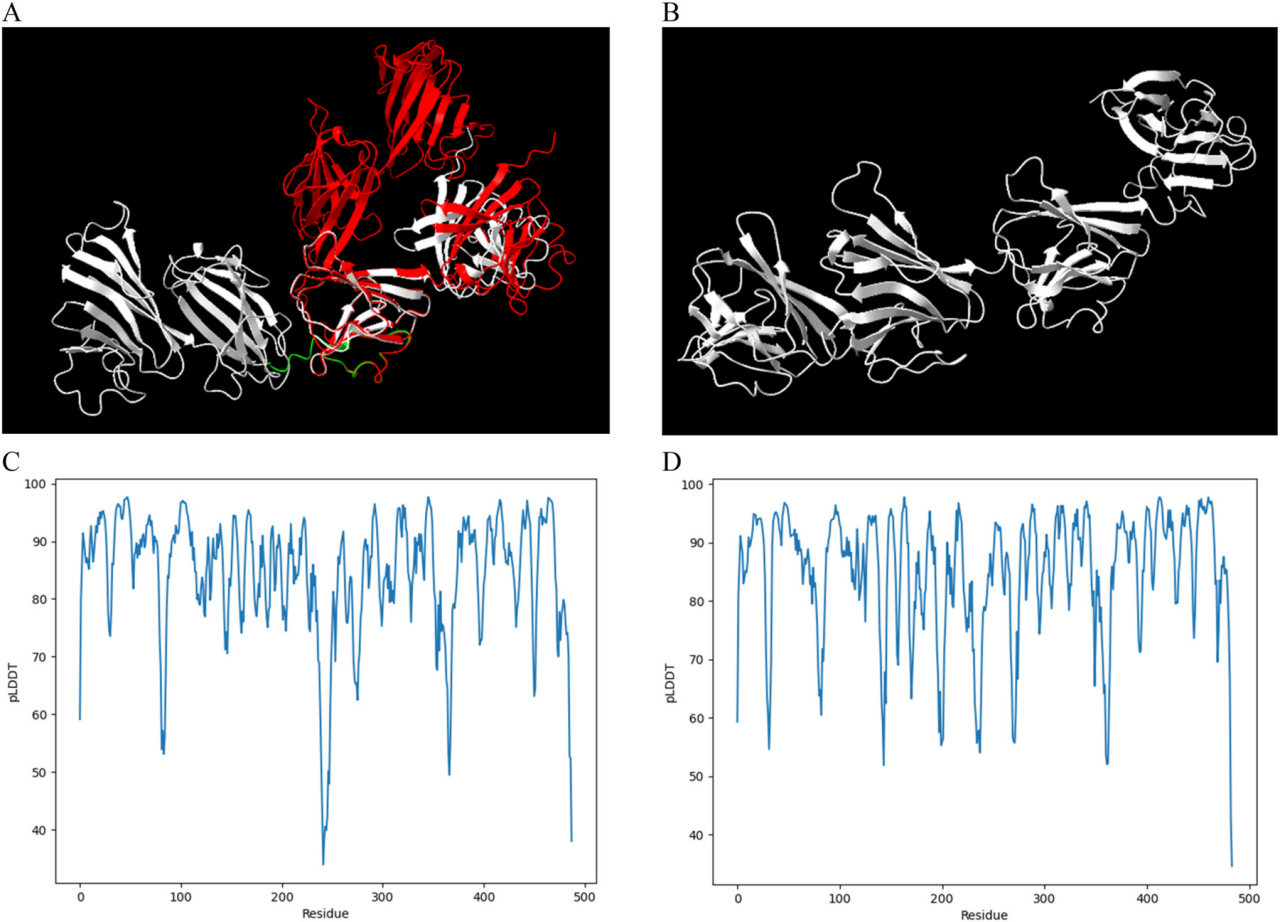
Protein structure prediction for ARM56 orthologs in *T. cruzi* and *T. brucei*. (A) ARM56 in *T. cruzi* (488 amino acids) was predicted using AlphaFold2 on the Galaxy server (white) and from AlphaFold DB (red). (B) ARM56 in *T. brucei* (484 amino acids) was predicted using AlphaFold on the Galaxy server (white). Protein structure predictions were visualized with ChimeraX. (C) Predicted LDDT in ARM56 in *T. cruzi* represented by pLDDT per residue, obtained by the model from Galaxy (model confidence score 84.42). Notably, lower pLDDT values were observed in the region from Leu239 to Ser267 (highlighted in green). (D) Predicted LDDT in ARM56 in *T. brucei* represented by pLDDT per residue, obtained by the model from Galaxy (model confidence score 85.10).

To conclude the visual inspection between the model’s outputs when applied to different species, the analysis was extended to *T. cruzi* and *T. brucei*. However, it is important to note that only ARM56 has orthologs, making it impossible to conduct a further inspection for ARM58. The comparisons for both proteins were carried out using the same model, AlphaFold2. This involved analysing predictions across various trypanosomatids to identify differences in the distribution of domains, as the structure of these domains was conserved. The proteins encoded by the genes TcCLB.506407.50, present in *T. cruzi*, and Tb927.10.2610 found in *T. brucei*, were employed for the purpose of analysing their differences in comparison to their ortholog ARM56 in *Leishmania*, as well as between different models. The sequences were sourced from the integrated database TryTripDB, considering the available information. Differences become apparent between the second and the third domain, with notable uncertainties present. This observation aligns with the lower values recorded in the predicted pLDDT plot within the range of amino acids 239 to 267 (Figure 8A and C). In the case of ARM56 ortholog in *T. brucei*, the predicted protein structure was obtained by running AlphaFold2 on the Galaxy server. However, no data was available for this strain in the AlphaFoldDB. Therefore, in this case, the comparison was conducted solely between the prediction from AlphaFold2 and uncertainties in specific regions can be observed in the predicted pLDDT plot (Figure 8B and D).

Variations between models depend significantly on the method and speed. Each predicted model comes along with the metrics, providing information about the confidence. Using different metrics for evaluation can make it difficult to compare the outputs. However, introducing structures predicted in several models and using other species or proteins with similar domains into the comparison could serve as a new approach to enhance our understanding of protein structure prediction, especially in cases where no previous data is available.

To summarize the observations obtained during the comparative analysis performed with ARM58 and ARM56, including orthologs in *T. cruzi* and *T. brucei*, RMSD values were calculated and measured in angstroms (Å). A comparison of protein structures for ARM58 was conducted using prediction models within the *Leishmania species* (Table 2). Within the *L. infantum* protein sequences, diverse RMSD values were observed: AlphaFold DB versus AlphaFold (Galaxy) showed a relatively low RMSD of 14.667 Å, while comparisons between trRosetta and RoseTTAFold resulted in a higher RMSD of 23.853 Å. Notably, the comparison of AlphaFold DB with RoseTTAFold exhibited the highest RMSD at 70.212 Å. Additionally, comparisons between different *L. species* revealed varying RMSD values, with *L. donovani* (AlphaFold Galaxy) versus *L. major* (AlphaFold Galaxy) displaying a lower RMSD of 8.173 Å compared to the higher RMSD of 31.669 Å between *L. donovani* (AlphaFold DB) and *L. major* (AlphaFold DB). These results highlight the variability in RMSD values influenced by the choice of prediction models and the comparisons among distinct protein sequences and species. Next, RMSD values were calculated for ARM56 and its orthologs, comparing protein structures across various prediction models (Table 3). In the *L. species*, comparisons revealed diverse RMSD values, with notable differences between prediction models such as AlphaFold DB, trRosetta, and RoseTTAFold. Additionally, comparisons among ColabFold models and different versions of ESMFold displayed varying RMSD values. Comparisons involving orthologs in *T. cruzi* and *T. brucei* demonstrated significant RMSD values. Furthermore, differences in protein sequence lengths influenced the number of pairs compared. These findings underscore the variability in RMSD values depending on the utilized prediction models and the comparisons made between different protein sequences and species.

**Table 2:** Calculation of the RMSD for ARM58 using a reference structure and a structure to match.

Reference structure		Structure to match		RMSD (517 pairs)
Protein sequence	Prediction model	Protein sequence	Prediction model	Angstrom
*L. infantum*	AlphaFold DB	*L. infantum*	AlphaFold (Galaxy)	14.667
*L. infantum*	trRosetta	*L. infantum*	RoseTTAFold	23.853
*L. infantum*	AlphaFold DB	*L. infantum*	trRosetta	47.066
*L. infantum*	AlphaFold DB	*L. infantum*	RoseTTAFold	70.212
*L. infantum*	ColabFold (I)	*L. infantum*	ColabFold (II)	7.340
*L. infantum*	AlphaFold DB	*L. infantum*	ColabFold (I)	15.711
*L. infantum*	AlphaFold DB	*L. infantum*	ColabFold (II)	12.659
*L. infantum*	ESMFold r4	*L. infantum*	ESMFold r8	12.070
*L. infantum*	AlphaFold DB	*L. infantum*	ESMFold r4	28.986
*L. infantum*	AlphaFold DB	*L. infantum*	ESMFold r8	35.632
*L. donovani*	AlphaFold (Galaxy)	*L. major*	AlphaFold (Galaxy)	8.173
*L. donovani*	AlphaFold DB	*L. major*	AlphaFold DB	31.669
*L. infantum*	AlphaFold (Galaxy)	*L. donovani*	AlphaFold (Galaxy)	15.486
*L. infantum*	AlphaFold (Galaxy)	*L. major*	AlphaFold (Galaxy)	21.086
*L. infantum*	AlphaFold DB	*L. donovani*	AlphaFold DB	21.165
*L. infantum*	AlphaFold DB	*L. major*	AlphaFold DB	21.224

**Table 3:** RMSD calculations for ARM56 and orthologs using a reference structure and matching structure.

Reference structure		Structure to match		RMSD (491 pairs)
Protein sequence	Prediction model	Protein sequence	Prediction model	Angstrom
*L. infantum*	AlphaFold DB	*L. infantum*	AlphaFold (Galaxy)	9.242
*L. infantum*	trRosetta	*L. infantum*	RoseTTAFold	19.971
*L. infantum*	AlphaFold DB	*L. infantum*	trRosetta	39.742
*L. infantum*	AlphaFold DB	*L. infantum*	RoseTTAFold	33.519
*L. infantum*	ColabFold (I)	*L. infantum*	ColabFold (II)	7.435
*L. infantum*	AlphaFold DB	*L. infantum*	ColabFold (I)	4.783
*L. infantum*	AlphaFold DB	*L. infantum*	ColabFold (II)	7.424
*L. infantum*	ESMFold r4	*L. infantum*	ESMFold r8	5.245
*L. infantum*	AlphaFold DB	*L. infantum*	ESMFold r4	44.793
*L. infantum*	AlphaFold DB	*L. infantum*	ESMFold r8	45.978
*L. donovani*	AlphaFold (Galaxy)	*L. major*	AlphaFold (Galaxy)	3.915
*L. donovani*	AlphaFold DB	*L. major*	AlphaFold DB	1.606
*L. infantum*	AlphaFold (Galaxy)	*L. donovani*	AlphaFold (Galaxy)	4.410
*L. infantum*	AlphaFold (Galaxy)	*L. major*	AlphaFold (Galaxy)	1.037
*L. infantum*	AlphaFold DB	*L. donovani*	AlphaFold DB	4.233
*L. infantum*	AlphaFold DB	*L. major*	AlphaFold DB	5.366
*T. cruzi*	AlphaFold DB	*T. cruzi*	AlphaFold (Galaxy)	49.304^(a)^
*T. cruzi*	AlphaFold (Galaxy)	*T. brucei*	AlphaFold (Galaxy)	14.802^(b)^

Different protein sequence lengths resulted in ^(a)^488 and ^(b)^483 pairs.

## 5 Discussion

The imprecise locations of atoms within a protein can be linked to flexible regions, given that a protein’s flexibility or rigidity is contingent upon the relative arrangement of its constituent atoms. The flexibility in specific protein sections can play a pivotal role in its function, facilitating conformational shifts necessary for interactions with other molecules or for fulfilling its biological role [32]. In this study, the predicted models of ARM58 and ARM56 effectively outlined the beta sheets present in each domain (Figure 2A and B). However, the precise atom positions across the four domains remain incompletely determined (Figure 2C and D). This indeterminacy might be indicative of flexibility, or it could signify uncertainty regarding exact positions. Extensive regions exhibiting a pLDDT value below 50 exhibit a strip-like pattern, hinting at a representation of disorder rather than actual structure [33].

Protein prediction models may produce structures that diverge from those generated by traditional models. Describing these models can be a formidable task; thus, it is crucial to correctly interpret the associated metrics. Model accuracy is assessed through metric such as pLDDT, PAE, or pTM scores, which necessitate thorough analysis by researchers. Some fast and reliable prediction methods, such as ColabFold [8], yield highly accurate protein structure predictions. However, discrepancies in these metrics (Table 1) can result in diverse outcomes (Figure 3). Certain models may excel when applied to specific sets of proteins. In the case of trRosetta and trRosettaX-Single, there are noticeable disparities in the confidence levels indicated by the pLDDT score (Figure 4). Each method generates distinct metrics to evaluate confidence; for example, RoseTTAFold, when used in conjunction with Robetta [10], provides predicted GDT (Global Distance Test) confidences. Different structure prediction systems yield varied models (Figure 5), adding complexity to the modelling process.

Protein structure modelling methods have the potential to yield structures that diverse from those produced by conventional methods. Elucidating these models can prove intricate, underscoring the importance of accurate metric interpretation. The precision of these models is assessed through metrics like pLDDT, PAE, or pTM score, which warrant careful analysis by researches. Certain quick and reliable prediction methods, like ColabFold [8] and ESMFold [11], supply accurate protein structure predictions. However, variations in the predictions lead to a range of outcomes. Diverse structure prediction systems yield contrasting, adding to the complexity of the modelling process. Remarkable differences in the overall spatial distribution were observed when using faster models such as ColabFold and ESMFold (Figures 3 and 6). In the case of ESMFold, the number of recycles could be increased to eight, although no significant variations were noted in this regard (Figure 6). A fusion of all the structures can offer supplementary insights into protein structures, particularly when there’s no pre-existing data from X-ray, NMR, or EM techniques. The typical approach to contrasting predicted and experimentally resolved structures involves the computation of the RMSD between the predicted and the experimental structures. The RMSD value gives the average deviation between the corresponding atoms of two proteins: the smaller the RMSD, the more similar the two structures.

There is no available structural data for ARM58 and ARM56, making a direct comparison of predictions unfeasible. Nevertheless, both proteins are present in other *Leishmania* species. As a result, the initial approach involved comparing the outputs from *L. donovani* and *L. major*. Variations and rotations can be observed in the regions between the domains in ARM58. A low confidence observed in the pLDDT scores might be linked to disorder [33]. However, the same distribution is observed when comparing ARM56 in the two different species (Figure 7). In this study, we have detected distinctions in the predictions generated by the tested models. By examining both the similarities and the confidence values through metrics, we identified variations that could be valid. Comparing metrics between models can be challenging because, in some cases, there are differences in how they are estimated. Furthermore, it has been observed that comparing with orthologs in other species could provide valuable information for determining the most likely ones. The variability in RMSD values, influenced by the choice of prediction models and the comparisons among different protein sequences and species, highlights the specific characteristics of the proteins and species under investigation (Tables 2 and 3). Moreover, it underscores the necessity for further refinement and validation of prediction methods to enhance the accuracy of protein structure comparisons across biological contexts.

ARM58 lacks orthologs in both *T. cruzi* and *T. brucei*, while ARM56 has orthologs in these species. Since we did not observe significant differences in the regions between the domains in ARM56, we decided to compare its predicted structure with the other two trypanosomatids. When we executed the AlphaFold2 model using Galaxy, the predictions exhibited a similar distribution. Although there was no prediction available for ARM56 in *T. brucei* within AlphaFold DB, one was available for *T. cruzi*. Notably, we observed a low pLDDT value between the second and the third domains in the prediction from AlphaFold2 executed in Galaxy, corresponding to a shift that becomes apparent when comparing the two outputs. The differences in protein structure predictions were primarily observed in the sequences between the domains. Both proteins exhibit a distribution of beta sheets that may be similar across each domain (Figure 8).

The proteins ARM58 and ARM56 are composed of four domains each, with unknown functions. The mechanism driving antimony resistance in *Leishmania* spp due to overexpression of both markers remains unknown. In this context, various protein prediction methods were employed to compare the anticipated structures of ARM58 and ARM56. Disparities exist between models, and each model can yield variations in different executions. Furthermore, alternate setups might also yield diverse results. By accurately simulating a range of transient protein complexes, end-to-end deep learning highlights potential avenues for future improvement, enabling dependable modelling of any protein–protein interaction that researches wish to explore [34]. Aspects like amino acid sequence composition or protein function could offer additional information. Besides predicting protein structures, AlphaFold2 also provides insights into the flexibility of residues or protein dynamics embedded in these structures [35]. The assortment of outcomes stemming from various protein prediction models suggest that differences between the models could unveil valuable insights regarding protein flexibility. Meticulous examination of these disparities is essential to decipher their significance and relevance in comprehending protein structure and function.

ARM58 and ARM56 comprise the four domains of unknown function (DUF1935), which are present in a range of bacterial and eukaryotic hypothetical proteins, along with the small myristoylated protein-1 (SMP-1) in *L. major*. In *L. major*, SMP-1 plays a crucial role in stabilizing the flagellar membrane and is essential for both flagella elongation and function [36]. Despite their prevalence, the precise functions of these domains remain undefined. The databases are continually expanding the number of predictions, yet there remains a need to validate these protein structures for a more comprehensive understanding of their configurations and folding patterns. By comparing diverse models that employ the same protein, along with closely related proteins found in orthologs, a broader range of structural variations can potentially be identified, which may be linked to various conformations or degrees of flexibility. This approach is essential to complement the experimental data acquired through laboratory research.

## 6 Conclusions and future work

Regions within proteins that lack well-defined structures introduce complexities in determining atom positions with precision. As a result, variations in these positions can yield a range of potential structures, underscoring the inherent flexibility of the protein. Additionally, the process of protein folding holds immense importance in drug development, as the protein’s three-dimensional configuration governs its functions and interactions with other molecules. Hence, it becomes crucial to identify which prediction imparts a more comprehensive understanding of the structure, enabling insights into diverse functional states or biologically relevant characteristics.

Understanding the generation of diverse outputs from deep learning models is essential. When complementary data from conventional prediction methods or experimental assays is available, additional insights can be gained. Often, lack of prior extensive investigation necessitates reliance on metrics for comprehending predictions, particularly when future experiments are in the pipeline. Future research might benefit from enhancing analysis by incorporating a broader and more significant range of proteins. Further analysis of the sequences should be performed to determine the accuracy of the predictions.
